# Perception of Dental Caries Risk and Preventive Strategies Among Orthodontic Patients: A Cross-Sectional Survey

**DOI:** 10.7759/cureus.80641

**Published:** 2025-03-16

**Authors:** Ayushi Singh, Jitendra Bhagchandani, Kavita Dhinsa, Sonali Saha, Vaibhav Vashishta, Amit K Singh

**Affiliations:** 1 Department of Orthodontics and Dentofacial Orthopedics, Sardar Patel Post Graduate Institute of Dental and Medical Sciences, Lucknow, IND; 2 Department of Pedodontics and Preventive Dentistry, Sardar Patel Post Graduate Institute of Dental and Medical Sciences, Lucknow, IND

**Keywords:** dental caries, fluorides, oral hygiene, orthodontic treatment, survey

## Abstract

Introduction: Orthodontic treatment significantly improves dental aesthetics, occlusal function, and overall oral health. However, maintaining optimal oral hygiene during treatment is challenging because of plaque accumulation around brackets, wires, and bands, which increases the risk of dental caries. Patient perceptions of caries risk and preventive strategies influence their adherence to oral hygiene measures. This study aimed to assess orthodontic patients’ perceptions of dental caries risk, adherence to preventive measures, and effectiveness of oral hygiene instructions provided by orthodontists.

Materials and methods: A cross-sectional survey was conducted at the Department of Orthodontics, India, for over 10 months (January 2024-October 2024). This study included 168 patients who had completed fixed orthodontic treatment and underwent bracket debonding. A structured, self-administered questionnaire was used to collect data on demographic details, perception of caries risk, adherence to oral hygiene measures, preventive strategies, and effectiveness of orthodontist-provided instructions. Chi-square tests were used for associations (p < 0.05, considered significant).

Results: One hundred twenty-eight (76.19%) participants reported difficulty in maintaining oral hygiene, and 132 (78.57%) experienced white spot lesions or early caries. Only 90 (52.38%) were aware of the role of fluoride in caries prevention, whereas 104 (62.50%) believed that orthodontists provided adequate oral hygiene instructions. After treatment, 144 (86.31%) felt confident in maintaining oral hygiene, but many emphasized the need for better patient education. Statistical analysis revealed a significant association (p = 0.001) between caries experience and fluoride awareness, oral hygiene adherence, and dietary modification.

Conclusion: Orthodontic patients face significant challenges in maintaining oral hygiene, with gaps in fluoride awareness and dietary adherence. Enhanced patient education, including fluoride use, dietary counseling, and behavioral reinforcement strategies, is essential. Structured oral hygiene guidance during orthodontic treatment is crucial to reduce caries risk and improve long-term oral health outcomes.

## Introduction

Orthodontic treatment plays a crucial role in enhancing patients' oral health, self-confidence, and overall quality of life by improving tooth alignment and occlusion. It not only contributes to better aesthetics but also helps to achieve functional efficiency of the dentition [1]. Despite its numerous benefits, orthodontic therapy presents significant challenges, particularly in maintaining optimal oral hygiene. The presence of orthodontic appliances, including brackets, wires, and bands, creates additional surfaces for plaque accumulation, which in turn increases the risk of dental biofilm formation [2]. This accumulation can lead to several oral health issues, including enamel demineralization, white spot lesions, dental caries, and gingival inflammation [3]. If left unaddressed, these conditions may progress to more severe periodontal problems, compromising both oral health and treatment outcomes [4].

Dental caries are one of the most prevalent oral health concerns associated with orthodontic treatment. The retention of plaque around orthodontic brackets provides an ideal environment for cariogenic bacteria, such as *Streptococcus mutans* and *Lactobacillus*, to thrive [5]. These bacteria metabolize dietary sugars and produce acids that demineralize enamel, leading to the development of carious lesions. Therefore, patients undergoing orthodontic treatment must adhere to stringent oral hygiene practices to minimize the risk of developing dental caries. Effective oral hygiene measures include regular toothbrushing with fluoride toothpaste, flossing with specialized interdental aids, use of antimicrobial mouth rinses, and adherence to dietary recommendations that limit sugar intake [6]. Despite the emphasis placed on oral hygiene by orthodontists, studies have shown that patient compliance with these preventive measures is often suboptimal, with adherence levels declining over time [7].

A crucial factor influencing patient compliance is the perception of dental caries risk and the effectiveness of preventive measures [8]. Although orthodontists provide comprehensive oral hygiene instructions at the beginning of treatment, patients may not fully understand the long-term implications of inadequate oral hygiene. Some patients may underestimate their vulnerability to dental caries or believe that the preventive measures recommended by their orthodontists are unnecessary or overly burdensome [7]. Others may struggle to integrate these practices into their daily routines because of lifestyle factors, lack of motivation, or misconceptions about oral health. This disconnect between professional guidance and patient adherence highlights the need for a deeper understanding of how orthodontic patients perceive their risk of dental caries and the preventive strategies available to them [6].

This study aimed to assess the perception of patients who underwent fixed orthodontic treatment for dental caries risk and prevention. The study objectives were to evaluate patients' perceptions of various preventive methods, such as proper tooth brushing techniques, dietary restrictions, oral hygiene adjuncts, and the use of fluoridated products. We further investigated the influence of oral hygiene instructions provided by orthodontists on patients’ understanding and adherence to caries prevention strategies. It seeks to explore how patients view different preventive measures and their effectiveness in reducing the risk of dental caries.

## Materials and methods

Study design

This cross-sectional survey was conducted in the Department of Orthodontics and Dentofacial Orthopedics, Sardar Patel Post Graduate Institute of Dental and Medical Sciences, Lucknow, India, over a period of 10 months from January 2024 to October 2024. This study was approved by the Institutional Ethical Committee, Sardar Patel Post Graduate Institute of Dental and Medical Sciences (FR/3/IEC/SPPGIDMS/2023) and followed the principles of the Declaration of Helsinki.

Selection criteria

The study employed a convenience sampling methodology, whereby all eligible individuals attending the department on the day of debonding were solicited to participate during the study's timeframe. The inclusion criteria for the study were male and female individuals aged 18 years and above who had finalized their fixed orthodontic treatment and had experienced bracket debonding on the survey date, as well as individuals who consented to participate in the study. Individuals with incomplete orthodontic treatment, those managed with removable appliances, or those who underwent fixed orthodontic treatment limited to a single arch were excluded from the study. Those with cognitive impairment and those who declined to participate were also excluded.

Data collection tool

The primary method of data collection was a structured self-administered questionnaire (see Appendix A) designed to assess patients' perceptions of dental caries risk and the effectiveness of preventive measures recommended during orthodontic treatment. The questionnaire was developed by a panel of experts including two orthodontists, one periodontist, one pedodontist, and one public health professional. Both the face and content validity were assessed to ensure the validity of the questionnaire. The assessment of face validity was conducted by convening a cohort of dental professionals to scrutinize the questionnaire for clarity, relevance, and suitability. Content validity was determined through the application of the Content Validity Index (CVI), wherein experts evaluated each item for relevance using a four-point Likert scale. Items with low CVI scores were revised or eliminated to improve the overall quality of the questionnaire. Additionally, construct validity was assessed by analyzing the questionnaire’s ability to measure the intended concepts, ensuring that it effectively captured the participants' perceptions of dental caries risk and prevention. Reliability testing was conducted to measure the consistency and stability of the questionnaire. Internal consistency was assessed using Cronbach’s alpha, with a value of 0.82 showing good reliability. To ensure test-retest reliability, the questionnaire was administered to a small subset of participants on two separate occasions approximately two weeks apart. The responses from both instances were compared using the intraclass correlation coefficient (ICC) to confirm the stability of the questionnaire over time, which yielded a value of 0.86.

Before the final implementation, a pilot study was conducted with a sample of 15-20 orthodontic patients who met the inclusion criteria and were not included in the study. Feedback from the pilot study participants was carefully analyzed and minor modifications were made to improve clarity and ensure that the questionnaire effectively captured the intended information. The results of the pilot study were not included in the main analysis but served as a crucial step in optimizing the study instrument.

Data collection

The questionnaire was administered to all eligible patients on the day of debonding. Each participant was informed about the study’s purpose, ensuring voluntary participation, and guaranteeing the anonymity of the responses. Written informed consent was obtained from all participants. The questionnaire gathered demographic details including sex, age, education level, and duration of orthodontic treatment. The questionnaire consisted of 10 questions to assess the perception of the participants regarding their caries experience during their orthodontic treatment, where the responses were measured using a three-point Likert scale, allowing for an analysis of varying levels of agreement. Participants’ perceptions of preventive behaviors for dental caries were examined by questioning their opinions on different approaches to avoiding tooth decay, such as maintaining proper oral hygiene, adhering to dietary restrictions, attending regular dental appointments, and reducing the intake of sugary foods and beverages. Participants were further asked two multiple-choice questions to determine whether they received guidance on essential oral hygiene practices during their orthodontic visits, including proper brushing techniques, dietary recommendations, use of oral hygiene adjuncts such as interdental brushes and floss, and use of fluoridated toothpaste and mouthwash.

Statistical analysis

Data analysis was performed using IBM SPSS Statistics for Windows, Version 23 (Released 2015; IBM Corp., Armonk, New York, United States). The Kolmogorov-Smirnov test was used to assess data normality, confirming a normal distribution. Categorical data are presented as frequencies and percentages. The chi-square test was used to assess the strength of the association between participants who experienced caries and those who did not. Statistical significance was set at p < 0.05, and a p-value less than 0.001 was set as highly statistically significant.

## Results

One hundred sixty-eight patients were provided the questionnaire with 54 (32.14%) males and 114 (67.86%) females with a mean age of 25.86 ± 5.32 years. The demographic distribution of participants indicated that the majority were females, which showed that more females were seeking fixed orthodontic treatment than males. The predominant age group seeking fixed orthodontic treatment was 18-20 years (n = 98, 58.33%), which indicated increased aesthetic awareness among the younger population. In terms of educational qualifications, the largest proportion of respondents held a bachelor's degree. Regarding the duration of orthodontic treatment, nearly half of the participants (n = 80, 47.62%) had undergone treatment for two years. Notably, a significant proportion of the respondents (n = 112, 66.67%) reported experiencing dental cavities during orthodontic treatment. This finding highlights a potential correlation between orthodontic treatment duration and increased risk of dental caries, emphasizing the need for enhanced oral hygiene education and preventive strategies during orthodontic therapy (Table 1).

**Table 1:** Baseline demographic details of the study participants (n = 168) Data is presented in the form of n (%).

Variables	Category	Frequency (n)	Percentage
Sex	Male	54	32.14
	Female	114	67.86
Age group	18-20 years	98	58.33
	21-23 years	18	10.71
	24-26 years	28	16.67
	> 26 years	24	14.29
Education level	Diploma or equivalent	42	25.00
	Bachelor's degree	80	47.62
	Postgraduate degree	46	27.38
Duration of orthodontic treatment	1 year	18	10.71
	2 years	80	47.62
	3 years	50	29.76
	> 3 years	20	11.90
Have you had any dental cavities during orthodontic treatment?	Yes	112	66.67
	No	56	33.33

The survey responses highlighted significant perceptions among patients regarding the impact of orthodontic treatment on their oral health, particularly concerning the risk of caries and oral hygiene maintenance. The majority of participants (n = 124, 73.81%) agreed that orthodontic treatment increased their risk of developing cavities, with the majority reporting difficulty in maintaining proper oral hygiene while wearing braces. Additionally, 132 (78.57%) participants experienced white spot lesions or early signs of cavities during treatment, reinforcing concerns regarding enamel demineralization in orthodontic patients. Despite these challenges, only 90 (52.38%) participants agreed that they were aware of the role of fluoride in caries prevention, suggesting a gap in the knowledge regarding preventive measures. Furthermore, while 132 (79.76%) participants agreed that they used additional oral hygiene aids regularly, 78 (46.43%) agreed that their dietary habits contributed to their increased risk of caries. Notably, 104 (62.50%) participants agreed that their orthodontists provided sufficient information on cavity prevention, indicating the need for enhanced patient education during treatment.

After treatment, 144 (86.31%) participants agreed that they felt confident in maintaining oral hygiene after bracket removal. Moreover, the same proportion recommended better education on caries prevention for future orthodontic patients, emphasizing the necessity of structured oral hygiene guidance during orthodontic care. These findings suggest that while patients recognize the challenges posed by orthodontic treatment, targeted educational interventions and preventive strategies are crucial for mitigating caries risk and improving overall oral health outcomes (Table 2).

**Table 2:** Participants responses according to Likert three-point scale as agree, neutral, and disagree Data is presented in the form of N (%).

S. No.	Questions	Agree		Neutral		Disagree	
		N	Percentage	N	Percentage	N	Percentage
1	I believe orthodontic treatment increased my risk of developing cavities.	124	73.81	16	9.52	28	16.67
2	I found it difficult to clean my teeth properly with braces on.	128	76.19	6	3.57	34	20.24
3	I was aware of the importance of fluoride in preventing cavities during treatment.	90	52.38	24	14.88	54	32.74
4	I regularly used additional oral hygiene aids (e.g., interdental brushes, mouthwash).	132	79.76	6	2.98	30	17.26
5	My orthodontist provided me with sufficient information about preventing cavities.	104	62.50	16	8.93	48	28.57
6	I think my dietary habits contributed to my cavity risk during treatment.	78	46.43	32	19.05	58	34.52
7	I experienced white spots or early signs of cavities while wearing braces.	132	78.57	6	3.57	30	17.86
8	I believe my oral hygiene routine was sufficient to prevent cavities during treatment.	106	64.29	16	8.93	46	26.79
9	Now that my braces are removed, I feel more confident about maintaining good oral hygiene.	144	86.31	16	8.93	8	4.76
10	I would recommend better education on caries prevention to future orthodontic patients.	144	86.31	6	2.98	18	10.71

Statistical analysis revealed a highly significant association between participants' experiences with caries during orthodontic treatment and their responses to various oral health-related questions (p = 0.001). Notably, participants who experienced cavities during treatment were significantly more likely to believe that orthodontic treatment increased their risk of developing cavities and found it difficult to clean their teeth properly. A highly significant disparity was observed in fluoride awareness (p = 0.001), with the majority of participants without caries recognizing the role of fluoride in cavity prevention. Similarly, those who did not develop caries were more likely to report sufficient oral hygiene practices, regular use of additional hygiene aids, and confidence in their oral care routine after treatment (p = 0.001).

Furthermore, most participants without caries perceived orthodontist-provided information on cavity prevention (p = 0.001). A significant difference was also observed in dietary influence perception (p = 0.001), with more participants experiencing cavities believing that their diet contributed to their risk. Overall, these findings underscore the critical role of patient education and preventive strategies for reducing caries incidence during orthodontic treatment. These statistically significant associations highlight the need for improved awareness of fluoride benefits, dietary modifications, and enhanced oral hygiene measures to mitigate caries risk in orthodontic patients (Table 3).

**Table 3:** Association between participants having caries experience and non-caries experience using chi-square test *p-value < 0.01: statistically significant, n indicates the number of participants, data is presented in the form of n (%).

Questions	Caries experience (n = 112 for yes, n = 56 for no)	Agree		Neutral		Disagree		Chi-stats	p-value
		n	Percentage	n	Percentage	n	Percentage		
I believe orthodontic treatment increased my risk of developing cavities.	Yes	68	60.71	16	14.29	28	25.00	29.81	0.001*
	No	56	100.00	0	0.00	0	0.00		
I found it difficult to clean my teeth properly with braces on.	Yes	72	64.29	6	5.36	34	30.36	26.25	0.001*
	No	56	100.00	0	0.00	0	0.00		
I was aware of the importance of fluoride in preventing cavities during treatment.	Yes	38	33.93	24	21.43	50	44.64	52.53	0.001*
	No	52	92.86	0	0.00	4	7.14		
I regularly used additional oral hygiene aids (e.g., interdental brushes, mouthwash).	Yes	76	67.86	6	5.36	30	26.79	22.91	0.001*
	No	56	100.00	0	0.00	0	0.00		
My orthodontist provided me with sufficient information about preventing cavities.	Yes	50	44.64	16	14.29	46	41.07	42.55	0.001*
	No	54	96.43	0	0.00	2	3.57		
I think my dietary habits contributed to my cavity risk during treatment.	Yes	28	25.00	32	28.57	52	46.43	63.02	0.001*
	No	50	89.29	0	0.00	6	10.71		
I experienced white spots or early signs of cavities while wearing braces.	Yes	76	67.86	6	5.36	30	26.79	22.91	0.001*
	No	56	100.00	0	0.00	0	0.00		
I believe my oral hygiene routine was sufficient to prevent cavities during treatment.	Yes	52	46.43	16	14.29	44	39.29	40.18	0.001*
	No	54	96.43	0	0.00	2	3.57		
Now that my braces are removed, I feel more confident about maintaining good oral hygiene.	Yes	88	78.57	16	14.29	8	7.14	14	0.001*
	No	56	100.00	0	0.00	0	0.00		
I would recommend better education on caries prevention to future orthodontic patients.	Yes	88	78.57	6	5.36	18	16.07	14	0.001*
	No	56	100.00	0	0.00	0	0.00		

**Figure 1:**
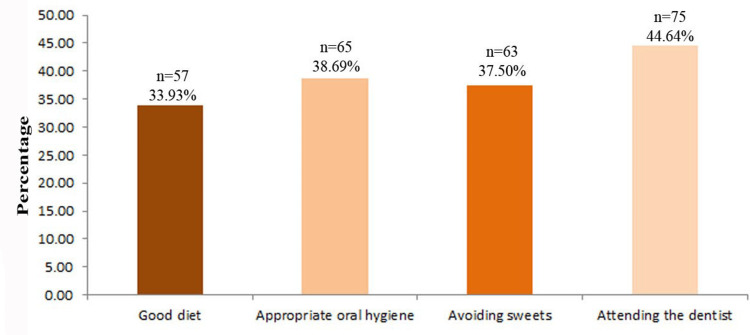
Oral hygiene practices among orthodontic patients This figure is derived from the data of the study.

Among the surveyed participants, 75 (44.64%) felt that regular orthodontic visits were crucial for maintaining oral health. This was followed by the maintenance of appropriate oral hygiene, emphasizing the importance of routine brushing and flossing. Additionally, avoiding sweets was considered an essential preventive measure by 63 (37.50%) participants, highlighting their awareness of the role of sugar in cavity formation. These findings suggest that, while participants recognize the importance of professional dental care and daily hygiene, there may be a need for greater awareness regarding the impact of dietary choices on oral health. Strengthening educational efforts regarding the role of nutrition in cavity prevention could further enhance comprehensive oral hygiene practices among orthodontic patients (Figure 1).

**Figure 2:**
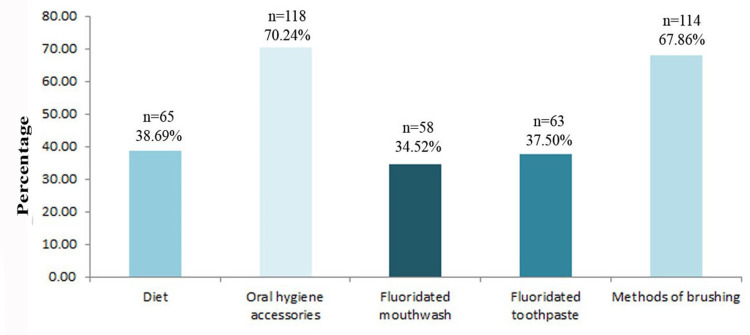
Information frequently provided by an orthodontist to their patient This figure is derived from the data of the study.

The information most frequently provided by an orthodontist to their patient was related to oral hygiene accessories and methods of brushing, indicating a strong emphasis on practical tools and techniques for maintaining oral health. In contrast, information regarding fluoridated toothpaste and fluoridated mouthwash is less commonly reported, suggesting a possible gap in patient education regarding the role of fluoride in cavity prevention. Similarly, only 65 (38.69%) participants recalled receiving information about the impact of diet on oral health, highlighting another potential area of improved patient education. These findings suggest that, while orthodontic professionals prioritize instruction on mechanical oral hygiene techniques, greater emphasis on dietary habits and fluoride use may be necessary to provide a more comprehensive approach to cavity prevention during orthodontic treatment (Figure 2).

## Discussion

The presence of fixed orthodontic devices, including brackets, bands, and wires, provides additional surfaces that promote plaque accumulation, thereby increasing the likelihood of enamel demineralization, development of white spot lesions, and incidence of dental caries [2]. The present study aimed to assess patients’ perceptions of dental caries risk and prevention during orthodontic treatment. The findings revealed that while patients acknowledged the challenges associated with oral hygiene maintenance during treatment, there were notable gaps in their knowledge and adherence to preventive strategies, particularly regarding fluoride use and dietary modifications.

Perception of increased caries risk during orthodontic treatment

A significant proportion of the participants perceived that orthodontic treatment increased their risk of developing dental caries. This perception aligns with existing literature, which suggests that orthodontic appliances create plaque-retentive sites that facilitate the accumulation of cariogenic bacteria such as *Streptococcus mutans* and *Lactobacillus* [9]. The mechanical complexity of fixed appliances makes it challenging to maintain adequate plaque control, thereby increasing the likelihood of enamel demineralization and white-spot lesions [2]. The duration of orthodontic treatment is associated with an increased risk of caries. Richter et al. [10] in their study found that 72.9% of patients developed at least one white spot lesion during the entire duration of orthodontic treatment, and 2.3% developed proper cavities.

Additionally, this study found a strong association between the participants' experiences with caries and their perceptions of oral hygiene difficulties. Participants who developed cavities during treatment were significantly more likely to report challenges in maintaining adequate oral hygiene than those who remained caries-free. Al-Melh et al. [11] conducted a study to detect cariogenic bacteria in the saliva of orthodontic patients using real-time polymerase chain reaction and found that *S. mutans* colony counts were significantly higher in orthodontic patients than in control subjects in one year of fixed orthodontic treatment. These results underscore the importance of reinforcing oral hygiene education for orthodontic patients to minimize plaque accumulation and prevent caries development. Similar results have been reported in other studies too [12,13].

Awareness and use of fluoride in caries prevention

Fluoride is widely recognized for its role in preventing dental caries by enhancing enamel remineralization and inhibiting demineralization [14]. However, the present study identified a gap in patient awareness regarding the role of fluoride in caries prevention. Only 52.38% of the participants acknowledged the importance of fluoride in reducing caries risk, indicating a significant deficiency in fluoride-related knowledge. This observation aligns with the research conducted by Akbar et al. [15], which revealed that only 40% of the participants employed topical fluorides. This study identified several impediments, including apprehension regarding potential overdosage, insufficient knowledge, and the prevailing notion that dental caries is a multifactorial condition that cannot be effectively mitigated solely through the application of topical fluorides.

Interestingly, a statistically significant difference was observed between participants who experienced cavities and those who did not, with fluoride awareness being significantly higher among the caries-free participants. This suggests that patients who are more knowledgeable about fluoride and its benefits may be more likely to adopt fluoride-based preventive measures, thereby reducing the risk of developing carious lesions. These findings align with those of Benson et al. [16], which highlight the necessity of incorporating fluoride education into orthodontic patient care.

Role of diet in caries risk and patient awareness

Diet plays a crucial role in the development of dental caries, particularly in patients undergoing orthodontic treatment. Sugary and acidic foods contribute to enamel demineralization by providing a substrate for cariogenic bacteria, which produce acid as a metabolic by-product [17]. The present study found that, while a significant number of participants acknowledged the importance of regular orthodontic visits for maintaining oral health, only 37.50% identified dietary modifications as an essential preventive strategy.

Moreover, a significant disparity was observed between participants who experienced cavities and those who did not, with the former being more likely to believe that their diet contributed to the risk of caries. This finding highlights a gap in dietary awareness and suggests that many orthodontic patients may not fully recognize the impact of sugar consumption on oral health. Similar findings were reported by Tufekci et al. [18], who found that orthodontic patients with high sugar intake were more prone to developing white spots and carious lesions. Providing patients with detailed dietary recommendations, including the reduction of sugary and acidic foods and beverages, may improve adherence to preventive strategies and reduce the incidence of caries in orthodontic patients.

Effectiveness of oral hygiene instructions provided by orthodontists

Orthodontic treatment success is highly dependent on patient adherence to the oral hygiene instructions provided by orthodontists. The study revealed that 62.50% of the participants believed their orthodontists provided sufficient information regarding cavity prevention. This suggests that, while orthodontists offer oral hygiene guidance, there may be a need for more structured and comprehensive educational interventions.

The most commonly provided information was related to oral hygiene accessories and brushing techniques, emphasizing mechanical plaque removal. However, less emphasis has been placed on fluoride use and dietary counseling. This discrepancy indicates that, while orthodontic professionals prioritize practical oral hygiene techniques, there is room for improvement in educating patients about the role of chemical and dietary interventions in caries prevention. Similar findings were reported by Berlin-Broner et al. [19], who found that 94% of participants agreed that they had been given adequate information on tooth brushing, whereas only 24.5% of patients reported that they had been given correct information on the use of fluoridated paste and mouthwash.

Patient adherence to preventive strategies

Despite the challenges associated with maintaining oral hygiene during orthodontic treatment, most participants reported feeling more confident in maintaining oral hygiene after bracket removal. This finding suggests that, while orthodontic treatment poses initial challenges, patients often develop improved oral hygiene habits over time.

Nevertheless, this research also revealed that patient adherence to preventive measures was inadequate throughout the course of active treatment. While a substantial majority of the study participants indicated the utilization of supplementary oral hygiene instruments, including interdental brushes and dental floss, a noteworthy segment continued to experience difficulties in sustaining optimal oral hygiene practices. This aligns with previous research indicating that adherence to oral hygiene recommendations declines over time, especially among adolescents [20].

Patient compliance is influenced by several factors, including motivation, perceived risk of dental caries, and perceived burden of preventive measures [8]. To improve adherence, orthodontists should adopt patient-centered approaches that involve motivational interviewing and behavioral reinforcement strategies. Research has demonstrated that individualized patient education, in conjunction with reinforcement provided by digital reminders or mobile applications, can significantly improve adherence to oral hygiene protocols among patients undergoing orthodontic treatment [21]. In a systematic review by Kommuri et al. [22], the role of antimicrobial mouthwashes during fixed orthodontic treatment is still debatable due to a lack of high-quality studies.

Clinical implications for orthodontic practice

The findings of this study have several important implications in orthodontic practice. First, there is a need for enhanced patient education regarding fluoride use and dietary modifications to improve caries prevention. Second, dietary counseling should be given more prominence in orthodontic care. Many patients may not fully recognize the impact of sugar consumption on caries risk, highlighting the need for structured dietary guidance. Providing patients with detailed nutritional recommendations and practical strategies for reducing sugar intake could contribute significantly to caries prevention. Last, patient adherence to oral hygiene recommendations can be improved through behavioral reinforcement strategies.

Limitations of the study

Notwithstanding its significant contributions to the field, this investigation was not devoid of certain constraints. First, the cross-sectional methodology constrains the capacity to ascertain causal connections between orthodontic intervention and perception of caries risk. Second, reliance on self-reported questionnaires may result in response bias, as respondents could potentially exaggerate or downplay their compliance with oral hygiene protocols. Finally, variables such as dietary habits, fluoride exposure such as water fluoridation, and personal oral hygiene practices, which could potentially affect caries risk outcomes, were not quantitatively assessed. Furthermore, though we have recorded the educational status of the participants, its effect on the prevalence of caries during fixed orthodontic treatment was not evaluated in our study. Subsequent longitudinal investigations are necessary to corroborate these findings.

## Conclusions

This study highlights the challenges orthodontic patients face in maintaining oral hygiene and their varying perceptions of caries risk and prevention. Significant gaps in fluoride awareness, dietary modifications, and adherence to oral hygiene practices were also observed. Patients with better preventive knowledge had a lower incidence of caries. Orthodontists should enhance patient education by emphasizing fluoride use, dietary counseling, and behavioral reinforcement strategies. Structured oral hygiene guidance during treatment is crucial for minimizing the risk of caries and improving long-term oral health outcomes in orthodontic patients.

## Appendices

Appendix A

Department of Orthodontics and Dentofacial Orthopedics and Department of Pedodontics and Preventive Dentistry

Survey Questionnaire

Perception of Dental Caries Risk and Preventive Strategies Among Orthodontic Patients: A Cross-Sectional Study

Part 1- Demographic details

Name-

Age-

Sex- Male/Female

Education- Diploma/Bachelor/Postgraduate

Duration of orthodontic treatment- 1/2/3/more than 3

Have you had any dental cavities during orthodontic treatment? - Yes/No

Part 2- Perception of caries risk

1-I believe orthodontic treatment increased my risk of developing cavities. Agree/Neutral/Disagree

2-I found it difficult to clean my teeth properly with braces on.  Agree/Neutral/Disagree

3-I was aware of the importance of fluoride in preventing cavities during treatment.  Agree/Neutral/Disagree

4-I regularly used additional oral hygiene aids (e.g., interdental brushes, mouthwash).  Agree/Neutral/Disagree

5-My orthodontist provided me with sufficient information about preventing cavities.  Agree/Neutral/Disagree

6-I think my dietary habits contributed to my cavity risk during treatment.  Agree/Neutral/Disagree

7-I experienced white spots or early signs of cavities while wearing braces.  Agree/Neutral/Disagree

8-I believe my oral hygiene routine was sufficient to prevent cavities during treatment.  Agree/Neutral/Disagree

9-Now that my braces are removed, I feel more confident about maintaining good oral hygiene.  Agree/Neutral/Disagree

10-I would recommend better education on caries prevention to future orthodontic patients.  Agree/Neutral/Disagree

Part 3- Preventive measures

1-In your opinion, which of those practices is a manner to avoid dental decay? "You can choose more than one answer"

Attending the dentist/ Appropriate oral hygiene/ A good diet/ Avoiding sweets.

2-What information did you get from orthodontist or dental assistant regarding oral hygiene care during orthodontic appointments? "You can choose more than one answer"

Methods of brushing/ Diet/ Oral hygiene accessories/ Fluoridated toothpaste/ Fluoridated mouthwash.
